# Sirtuin Evolution at the Dawn of Animal Life

**DOI:** 10.1093/molbev/msac192

**Published:** 2022-09-06

**Authors:** David A Gold, David A Sinclair

**Affiliations:** Department of Earth and Planetary Sciences, University of California, Davis, Davis, CA; Harvard Medical School, Boston, MA

**Keywords:** sirtuin, PNC1, NAMPT, animals, gene evolution

## Abstract

Sirtuins are a family of proteins that protect against cellular injury and aging; understanding their evolution should reveal fundamental mechanisms governing longevity. “Early-branching” animals such as sea sponges and jellyfish have been understudied in previous analyses of sirtuin diversity. These organisms not only hold important positions at the base of the evolutionary tree, but also have unique aging dynamics that defy convention, such as quasi-immortality and high regenerative capacity. In this study, we survey the evolution of sirtuin proteins in animals, with a focus on the oldest living lineages. We describe previously unrecognized expansions of “Class IV” and “Class I” sirtuins around the origin of animals, raising the number of sirtuin families in the last common ancestor to at least nine. Most of these undescribed sirtuins have been lost in vertebrates and other bilaterian animals. Our work also clarifies the evolution of PNC1 and NAMPT enzymes that carry out the rate-limiting step in sirtuin-related NAD^+^ biosynthesis. The genes for PNC1 and NAMPT enzymes were both present in the first animals, with the genes being lost a minimum of 11 and 13 times, respectively, over the course of animal evolution. We propose that species with these ancestral gene repertoires are ideal model organisms for studying the genetic regulation of animal longevity and will provide clues to increasing longevity in humans.

## Introduction

**Fig. 1.**
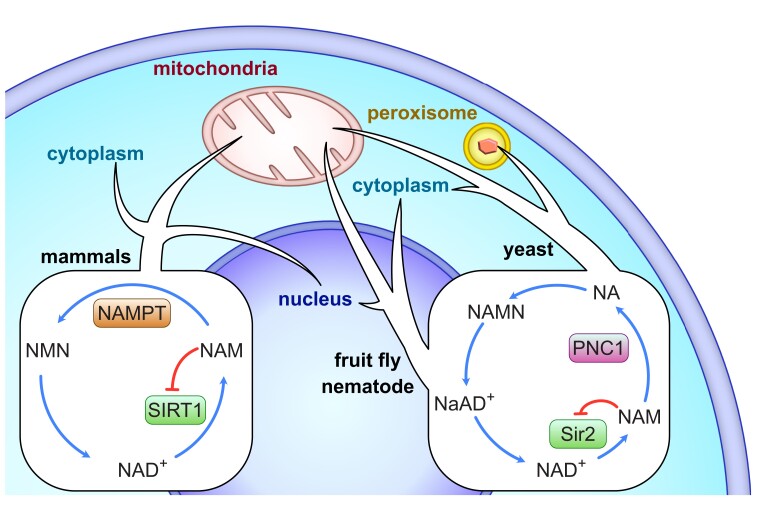
Overview of the different sirtuin/NAD^+^ pathways in model organisms, including mammals, fruit flies (*Drosophila melanogaster*), nematodes (*Caenorhabditis elegans*), and yeast (*Saccharomyces cerevisiae*). Arrows indicate the regions of the cell where sirtuin activity has been described in each organism.

Sirtuins are a family of lysine deacylases that use and respond to nicotinamide adenine dinucleotide (NAD^+^), a cofactor central to cellular metabolism and DNA repair. Targets of sirtuin activity include regulators of the epigenome (e.g., histone H3 K9 acertylation), DNA repair (e.g., p53), inflammation (e.g., NF-κB), and energy metabolism (e.g., PGC-1alpha; Choi and Mostoslavsky 2014; Mei et al. 2016). The first described sirtuin gene—named *silent information regulator 2* or *sir2*—came from the yeast *Saccharomyces cerevisiae*, and regulates cell aging through epigenetic gene silencing and nucleolar rDNA stability (Sinclair et al. 1997; Kaeberlein et al. 1999). A large body of work implicates sirtuins in similar processes in mammals, leading to the hypotheses that the gene could function as a conserved regulator of aging (Sinclair 2005). Yeast *sir2* and its mammalian homolog, SIRT1, perform these regulatory activities through protein deacylase activity, breaking NAD^+^ into nicotinamide (NAM) and fusing the acetyl moiety to adenine ribose in the process (Sauve and Youn 2012). NAM inhibits further sirtuin activity through negative feedback but can be converted back into NAD^+^ through the enzymes nicotinamide phosphoribosyltransferase (NAMPT) and/or pyrimidine nucleotide carrier 1 (PNC1; Bitterman et al. 2002). While NAMPT is found in mammals and some bacteria, PNC1 is found in model yeast, fruit flies, and nematodes (fig. 1), suggesting the canonical sirtuin pathway has undergone lineage-specific evolution. Additionally, most eukaryotes have multiple sirtuin genes, demonstrating a complex evolutionary history of gene duplication events, gene losses, and horizontal gene transfers (HGTs; Frye 2000, 2006). Today >15,000 sirtuins have been identified in over 6,000 species spanning all domains of life. These various sirtuin homologs have evolved a range of enzymatic functions that respond to NAD^+^, collectively controlling a suite of biological processes that connect metabolism to longevity. Reconstructing the evolutionary history of sirtuin biosynthesis is a crucial step to understanding aging dynamics in eukaryotes.

A universal taxonomy for sirtuins is improbable, but there is value in untangling their evolutionary history. Generating a taxonomic system for genes runs into the same troubles as classifying species; in both scenarios, we attempt to force a continually branching, evolutionary process into a hierarchical framework. Animal sirtuins have traditionally been divided into four “classes” and seven “families”: Class I (with families SIR1, SIR2, SIR3), Class II (SIR4), Class III (SIR5), and Class IV (SIR6, SIR7; Frye 2000). This system dictates which genes represent orthologs—or genes that arose through speciation events—and which are paralogs—or genes that arose through duplication events. However, studies starting with different species have produced different sets of orthologs and paralogs. For example, one recent study argued for an eighth paralog (“SIRT3 like”) present in fish and amphibians (Opazo et al. 2020). Such distinctions between orthologs and paralogs are not merely semantic; they determine how we ought to interpret functional data between model organisms. For example, yeast *sir2*, mammalian *Sirt1*, and nematode *sir-2.1* are all implicated in longevity. If these genes are orthologs, as currently hypothesized, it is probable that the last common ancestor of animals and fungi used this gene to regulate cell aging. By contrast, if they are paralogs, then *sir2*, *Sirt1*, and *sir-2.1* cannot be traced to a single gene in the last common ancestor and at least one, or perhaps all, of these genes changed function over time to regulate aging. There is only one evolutionary history that correctly describes the distribution of sirtuin genes, but the assignment of orthologs versus paralogs shifts depending on the group of organisms being studied (supplementary fig. S1, Supplementary Material online). It is therefore critical to consider the group of organisms one wants to target before developing a taxonomic nomenclature.

Here, our goal is to generate a sirtuin taxonomy consistent with the origin and early diversification of animals. This requires a focus on “early-branching” animals—those living groups that were first to “branch off” the animal tree of life. The early-branching animals include the phyla Porifera (sea sponges), Ctenophora (comb jellies), Placozoa (amoeba-like animals), and Cnidaria (sea anemones, corals, jellyfish, and hydras). The term early-branching is preferable to “ancestral” or “primitive,” as the latter terms imply that the living members of these phyla are unchanged from the first animals that evolved hundreds of millions of years ago. In truth, every living species contains a mosaic of ancestral and derived traits, but careful comparisons between early-branching species can provide insights into the first animals. Indeed, these clades represent the living descendants of the first animals, as they experimented with multicellularity, nervous systems, guts, and the basic biological processes that define the “higher” species (collectively known as the Bilateria).

The correlation between sirtuin evolution and animal origins is intriguing, but few studies have included more than one early-branching animal, if any. One study included multiple early-branching animals and concluded that all seven sirtuin families were present in the last common ancestor of animals (Greiss and Gartner 2009). However, this study did not sample sponges or ctenophores—the oldest living animal groups—and had a small sampling of cnidarians and placozoans. Previous studies of early-branching animal genomes have revealed an unexpectedly high amount of cryptic diversity, meaning that phyla must be well-sampled to make a confident assessment of their gene repertoires. Elucidating the evolution of sirtuins in the context of animal origins, therefore necessitates a study focused on early-branching groups.

In addition to evolutionary considerations, early-branching animals also provide important insight into longevity and senescence. Many early-branching species exhibit aging processes that defy expectations, making them ideal candidates to study the biology of longevity (Petralia et al. 2014). Most early-branching animals exhibit one or more forms of asexual reproduction, meaning cell lines making up the “adult” organism can continue, presumably indefinitely, without the cellular resetting that occurs during zygote formation. Isotopic data from the skeleton of a deep-sea sponge *Monorhaphis chuni* gave an age of ∼11,000 years, making it the oldest living animal known (Jochum et al. 2012). Corals are made up of hundreds of clonal polyps, which can collectively live for 500 years or more (Montero-Serra et al. 2018). Some *Hydra* polyps demonstrate no evidence of aging, while in other species the aging process can be turned on or off using environmental or genetic triggers (Boehm et al. 2012). Many jellyfish go through a dual lifecycle, including a clonal, potentially immortal polyp stage and a medusa stage that demonstrates senescence (Gold and Jacobs 2013). In most of these cases, the cell lines making up individuals or populations have not been studied in enough detail to determine the extent to which these organisms are truly immortal, or by what mechanisms they avoid the rapid senescence seen in most comparably sized invertebrates. Understanding how sirtuins function in these organisms will be an important part of elucidating the genetic regulation of animal longevity.

## Results

**Fig. 2.**
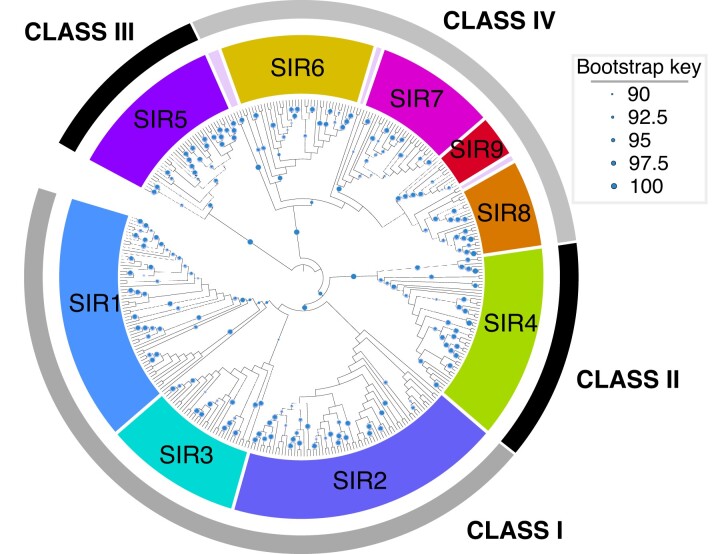
Phylogeny of sirtuin genes. This phylogeny was generated using IQ-TREE, and then reconciled with the species tree using NOTUNG (Chen et al. 2015; Nguyen et al. 2015). Circles indicate nodes with Ultrafast Bootstrap Approximation values ≥90. See Materials and Methods for details.

**Fig. 3.**
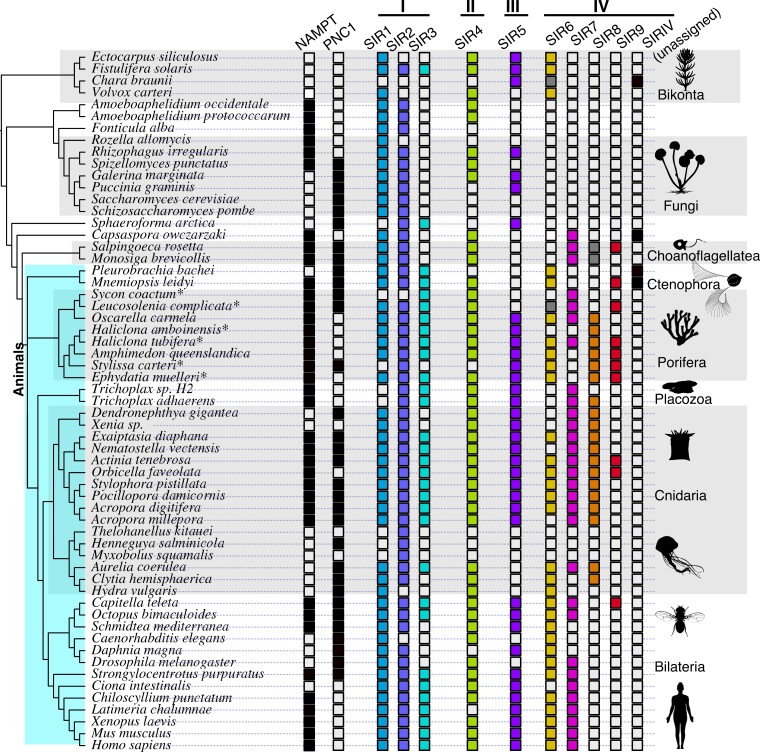
Distribution of NAMPT, PNC1, and sirtuins across our data set. Asterisks next to species names imply the data come from a transcriptome as opposed to a genome, meaning absence of genes should be taken with caution. Gray squares indicate that one or more sirtuins from this taxon might be members of the relevant clade, but the assignment lacked statistical support (rapid bootstrap values ≥90).

Our sirtuin phylogeny—which combines deep sampling of early-branching animals with a diverse set of opsithokonts (animals, fungi, and their relatives)—is broadly consistent with previous analyses. We recover the four major classes as well as the seven previously recognized families (fig. 2; detailed tree in supplementary fig. S2, Supplementary Material online). The distribution of sirtuins across our tree suggests a complicated history of gain and loss (fig. 3). No single family of sirtuins appears essential, although some show greater conservation than others. Our results confirm various hypotheses from previous studies. For example, earlier studies have identified a clade of fungal sirtuins—including yeast *HST3* and *HST4*—that are part of “Class I” but have not been assigned to any particular family (Greiss and Gartner 2009). We also recover the clade as a member of Class I, and in our analysis are able to assign it to the SIR1 family. Our results also support a *SIR3*/*SIR3*-like division in non-amniote vertebrates, and suggest that the gene duplication responsible for these paralogs is vertebrate specific (Opazo et al. 2020). Most relevant, our results support the hypothesis that all seven currently recognized sirtuin families were present in the first animals (Greiss and Gartner 2009).

### Multiple Classes IV and I Sirtuins Evolved Around the Time of Animal Diversification and were Lost in most Bilaterians

**Fig. 4.**
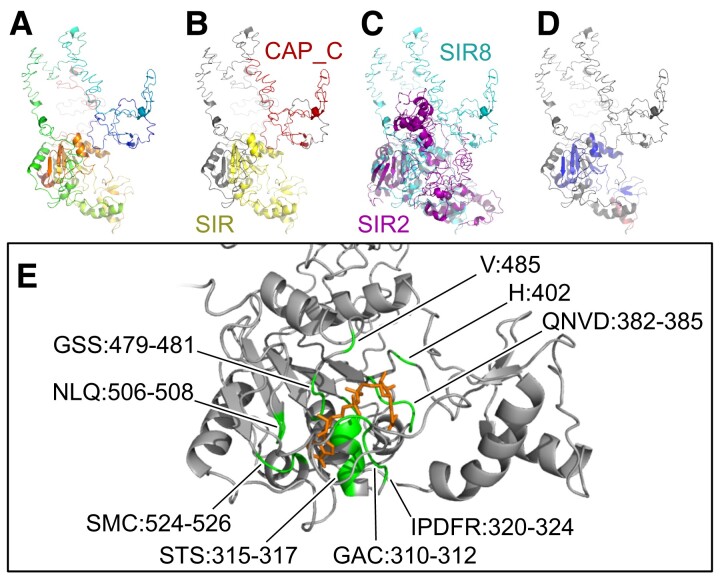
Modeling of the SIR8 protein. Modeling was performed in I-TASSER based on protein XP_011406188 from the sea sponge *Amphimedon queenslandica*. (*A*) Predicted structure of SIR8. (*B*) The same protein with the two conserved domains highlighted. (*C*) Alignment of SIR8 and the *Saccharomyces cerevisiae* SIR2 protein. (*D*) SIR8 colored by the root-mean-square deviation of atomic positions (RMSD) based on the alignment with SIR2. SIR2 has been removed for clarity. Dark blue is a good alignment, higher deviations are in orange/red, unaligned residues are white. (*E*) Predicted NAD ligand-binding-site residues in SIR8. NAD is semi-transparent, visualized using a Gaussian volume representation. Images created in Open-Source PyMOL.

With our detailed analysis of early-branching animals, we double the number of known Class IV sirtuin families in animals. The previously described Class IV sirtuins—SIR6 and SIR7—both predate animals; SIR7 is found in other holozoans, and Sir6 has previously been described in plants and diatoms (Greiss and Gartner 2009). However, we identify two new Class IV family members, which we name SIR8 and SIR9. SIR8 is particularly common in sponges, some corals, and jellyfish, many of which also have copies of SIR6 and SIR7. We identified a ∼9 amino acid motif unique to SIR8 (supplementary fig. S3, Supplementary Material online), and about half of our SIR8 sequences have a “CAP-C” protein domain upstream of the typical “Sir2” domain (supplementary table S1, Supplementary Material online). While gene-tree/species-tree reconciliation supports the hypothesis that SIR8 also exist in choanoflagellates (fig. 3), we note that these choanoflagellate sirtuins lack the CAP-C domain and the SIR8-specific amino acid motif (supplementary fig. S3, Supplementary Material online). The CAP-C domain (Pfam ID: PF08603) is normally found in cyclase-associated proteins, where it plays a role in actin binding (Hubberstey and Mottillo 2002). We modeled the structure of SIR8 using I-TASSER (Yang et al. 2015) to test what impact this protein domain fusion might have (fig. 4). Despite the increased size from the extra domain, modeling suggests that the sirtuin-specific domain in SIR8 remains structurally similar to the well-studied SIR2 homolog, particularly in the region where deacetylase activity occurs (fig. 4*C*and*D*). Consistent with this similarity in structure, I-TASSER predicts that SIR8 is capable of NAD ligand binding and hydrolase activity (fig. 4*E*). We did not find evidence for ligand binding or enzymatic activity in the region around the CAP-C domain. What role this additional sirtuin plays in the biology of early-branching animals is unclear, but our study provides many candidate organisms for further exploration. Sillouhettes were collected from PhyloPic: sponge sillouhette made by Mali'o Kodis, photograph by Derek Keats; placozoan silhouette by Oliver Voigt.

In addition to SIR8, we identified another undescribed Class IV gene, which we call SIR9. Similar to SIR8, SIR9 has a ∼25 amino acid insertion that is distinct from other sirtuins (supplementary fig. S4, Supplementary Material online). SIR9 genes are present in some choanoflagellates, ctenophores, sponges, and cnidarians, suggesting the gene arose just prior to the origin of animals and was retained in many early-branching lineages. Surprisingly, the gene is also found in the annelid worm *Capitella* (fig. 3). Since this was the only bilaterian in our data set containing the protein, we queried National Center for Biotechnology Information (NCBI) to see if any other animals had a sirtuin with the conserved SIR9 motif. Indeed, we recovered putative SIR9 homologs from multiple gastropod and bivalve molluscs, suggesting the gene was present in the last common ancestor of molluscs and annelids (clade Lophotrochoza). The loss of SIR9 across many major animal lineages, including cnidarians, deuterostomes, and ecdysozoans, appears the most parsimonious explanation for our results.

**Fig. 5.**
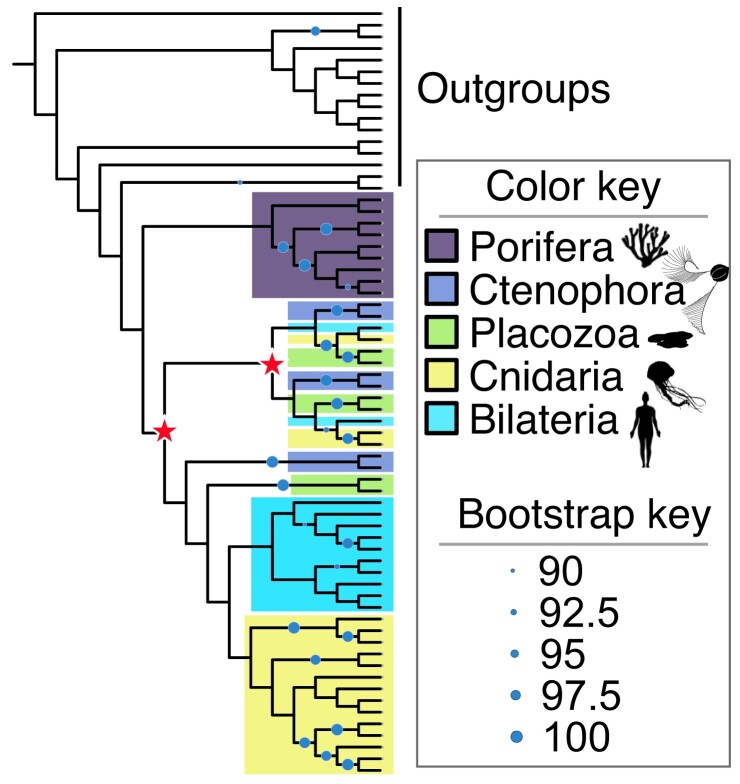
Phylogeny of SIR2 proteins. These results are from an independent analysis of SIR2 proteins separate from the rest of the data set (see Materials and Methods for details). Circles indicate nodes with Ultrafast Bootstrap Approximation values ≥90. Stars indicate putative gene duplication events. Note that this tree assumes that Porifera represents the earliest-branching clade of animals. A ctenophore-first phylogeny results in a different, more complex pattern of gene duplication events. See the GitHub repository for detailed results.

In addition to SIR8 and SIR9, we found evidence for multiple SIR2 (Class I) paralogs in early-branching animals. SIR2 was the only sirtuin family we could not constrain with statistical support (i.e., rapid bootstrap value >90), although gene-tree/species-tree reconciliation supports its monophyly (fig. 2, supplementary fig. S2, Supplementary Material online). To improve resolution of the SIR2 topology, we re-ran the analysis performed on the entire sirtuin data set to SIR2 proteins only (see Materials and Methods). This added a moderate number of additional characters to the gene alignment (29 amino acids), which unfortunately failed to increase statistical support for anything but the most derived nodes. Our results are summarized in fig. 5, with a more detailed tree in supplementary fig. S5, Supplementary Material online. Gene-tree/species-tree reconciliation suggests that two rounds of gene duplication occurred following the separation of sponges from the other animals, although the paralog clades are poorly supported and the exact timing of these events changes depending on the species tree used (see Materials and Methods). Because we cannot define SIR2 subclades with statistical confidence, we do not attempt to name them in this study. Despite this uncertainty, our results demonstrate that many early-branching animals contain as many as three copies of the SIR2 protein, including all placozoans and ctenophores sampled. We are confident that the relationships of these SIR2 paralogs will be resolved as more genomes from early-branching animals become available, and they provide another line of evidence that sirtun dynamics were an important part of early animal evolution.

Based on these results, the last common ancestor of animals had at least nine sirtuins from four classes: Class I (SIR1, SIR2, and SIR3), Class II (SIR4), Class III (SIR5), and Class IV (SIR6, SIR7, SIR8, and SIR9). Both SIR8 and SIR9 likely arose through gene duplications just prior to the split of animals from their closest living relatives, the choanoflagellates. There were also at least two SIR2 gene duplication events early in animal evolution, although the exact timing of these events and the assignment of paralogs is currently unclear. Future work resolving these SIR2 paralogs could easily raise the ancestral sirtuin repertoire to 10 or 11.

### Little Evidence for Horizontal Gene Transfer or Chimeric Genes in Animal Sirtuins

The HGT of sirtuins from distantly related organisms could have played an important role in the expanded number of animal paralogs. In our first round of tree building, we recovered one clade of sirtuins from a range of distantly related animals that did not fall into any known sirtuin family. Reciprocal BLAST analysis of these genes demonstrates that they are all most similar to microbial sequences. Most of the species in this clade either have no genome or a draft genome, which makes it difficult to determine whether these unusual sirtuins truly come from the organism in question. The taxon in this clade with the best annotated genome is the sea urchin *Strongylocentrotus purpuratus*; an NCBI BLASTP search using the *St. purpuratus* sirtuin as a query only revealed bacterial hits, meaning this gene is not present in any other echinoderm and is likely the result of contamination. We also discarded one additional SIR5-like sequence from the sponge *Stylissa*, which did not clade with the other sea sponge SIR5 sequences and whose reciprocal BLAST hit was to the bacteria *Thermococcus*. We note that the *Stylissa* transcriptome included an additional SIR5 that clades with sea sponges, meaning excluding this sequence has no impact on the presence/absence of sirtuins in this taxon. The sirtuins excluded from the final analysis are listed in supplementary table S2, Supplementary Material online and annotated in supplementary fig. S6, Supplementary Material online. While we provide this data as a resource for future research, we suspect that these genes are more likely the result of contamination than genuine HGTs. Based on the data here, it does not appear that HGT events played an important role in the evolution of animal sirtuins, but better taxon sampling might demonstrate that some of these putative HGT events are genuine.

In addition to HGT, the shuffling of conserved domains and/or the production of chimeric sequences also has a profound impact on gene evolution, and can complicate attempts to place genes in a phylogenetic framework. In addition to the CAP-C domain described earlier in some SIR8 sequences, supplementary table S1, Supplementary Material online provides some additional conserved identified in other sirtuins. There is a domain of unknown function (DUF592) found in some fungal SIR1 sequences, but to the best of our knowledge this domain is not found in other genes. There are some other putative domain fusions—including domains for radical SAM, oxysterol binding, and molybdenum cofactor synthesis—but each of these was recovered from a single sequence, and could easily be the result of improper gene annotation from the respective genomes. Again, future work might demonstrate interesting cases of chimeric evolution for sirtuins, but we found little evidence for it in our data set.

### NAMPT and PNC1 were both Present in Early Animals and Subsequently Lost

**Fig. 6.**
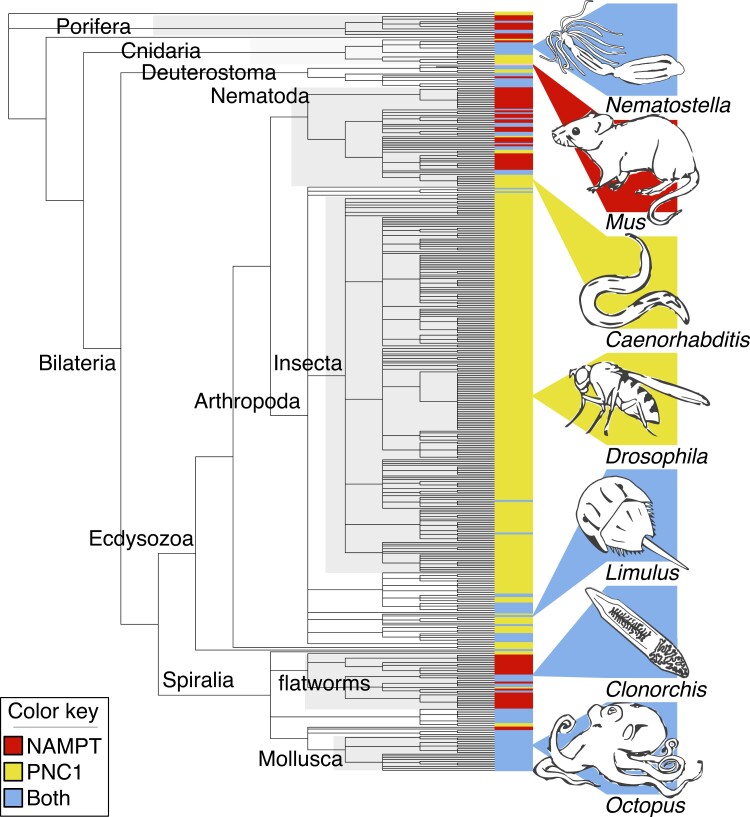
Distribution of NAMPT and PNC1 across animals. This study was restricted to invertebrate animals, but the placement of vertebrates is noted in the tree with the addition of *Mus*.

While the presence of both PNC1 and NAMPT has been demonstrated in multiple invertebrates (Gossmann et al. 2012; Carneiro et al. 2013; Bockwoldt et al. 2019), there remains a persistent notion that they regulate mutually exclusive pathways. Our study confirms that both genes were present in the last common ancestor of animals, demonstrated by their retention in living choanoflagellates and some sponges and ctenophores (fig. 3). Given the complex pattern of gain and loss seen in fig. 3, we did an additional analysis examining all invertebrate NAMPT and PNC1 genes in the NCBI protein database. Our results, summarized in fig. 6 (full results in supplementary fig. S7, Supplementary Material online), show that both proteins are commonplace in many animal clades, including calcareous sponges, hexacorals, decapod crustaceans, lophotrochozoans, and echinoderms. Taking a conservative approach, we estimate that PNC1 has been lost at least 11 times in animal evolution, and NAMPT has been lost at least 13 times (supplementary fig. S7, Supplementary Material online); these are almost certainly underestimates of the true number of losses.

## Discussion

The goal of this study was to elucidate the evolution of sirtuins and their cofactors from the vantage of early animals. Our results demonstrate that this history is marked by an early, previously unrecognized expansion followed by a high level of lineage-specific loss. The last common ancestor of animals had at least nine sirtuins from four classes: Class I (SIR1, SIR2, and SIR3), Class II (SIR4), Class III (SIR5), and Class IV (SIR6, SIR7, SIR8, and SIR9). SIR9 likely arose through a gene duplication from SIR6 or SIR7 just prior to the split of animals from their closest living relatives, the choanoflagellates. An additional gene duplication led to the evolution of a new family, SIR8, around the same time or after the animal/choanoflagellate split. These two families have not been previously described because they have been lost in most living animals. Based on our sampling, SIR8 appears to have been independently lost in ctenophores, calcareous sponges, placozoans, and bilaterians. Similarly, SIR9 was lost in many sea sponges, corals, and jellyfish, and must have also been lost over a dozen times in bilaterians, given its presence in some annelid worms and molluscs. Such high levels of gene loss is commonplace in animal evolution (Albalat and Cañestro 2016; Guijarro-Clarke et al. 2020), and our analysis on sirtuins adds to this growing list of examples. Given the high level of gene loss, the early expansion of Class IV sirtuins would have been difficult to capture without a diverse sampling of early-branching animals.

The history of the sirtuin-related proteins NAMPT and PNC1 is also marked by repeated, lineage-specific loss. These two proteins mediate different NAD salvage pathways, which are often thought of as equivalent and mutually exclusive despite extensive research showing both genes are present in a variety of organisms (Gazzaniga et al. 2009; Gossmann et al. 2012; Bockwoldt et al. 2019; Audrito et al. 2020). This study is not the first to note that some invertebrates have both proteins, meaning both must have been present in the last common ancestor of animals, but we reveal how extensive and complex the pattern of loss has been. Several animal clades show repeated loss of one of the two proteins; for example, NAMPT has been lost multiple times independently in the arthropods, while sponges and flatworms almost always abandon PNC1. This suggests the loss of one of these two proteins is not random. As a clade, Nematodes appear unusual for the frequency at which NAMPT or PNC1 is lost, which could be related to the hosts these largely parasitic animals inhabit. Some of these gene losses are ancient: the loss of vertebrate PNC1 likely goes back to their last common ancestor ∼457–636 million years ago (Ma), while the absence of NAMPT in the fruit fly *Drosophila* probably stems from a loss that, at minimum, occurred around the origin of the Diptera ∼72–25 Ma and might trace back to the last common ancestor of insects ∼405–521 Ma (Benton et al. 2015; Wolfe et al. 2016). Other losses are much more recent; while NAMPT is missing from nematodes in the genus *Caenorhabditis*, it is retained in the closely related *Diploscapter pachys*. The absence of NAMPT in fly and nematode model organisms is therefore a coincidence, resulting from two distinct evolutionary events. And as intriguing as these many cases of loss are, there are many animal lineages that have retained both proteins over 550+My of evolution. Our results lead to the paradoxical conclusion that NAMPT and PNC1 are not functionally synonymous, yet one or the other is often expendable.

As expected, there is no obvious correlation between longevity and the number of sirtuins a species has, the presence/absence of a particular sirtuin family, or the presence/absence of NAMPT/PNC1. However, there are some intriguing patterns, particularly in the early-branching animals. Many early-branching animals retain both NAMPT and PNC1 and could be good candidates for studying their biological functions when both are present. Emerging model organisms with both proteins include the ctenophore *Mnemiopsis*, the sea anemone *Nematostella*, and the coral *Acropora*. Similarly, SIR8 and SIR9 are largely restricted to early-branching animals. Emerging model organisms with SIR9 include the sponge *Amphimedon* and the sea anemone *Actinia*, while SIR8 could be studied in *Nematostella*, *Amphimedon*, and/or the jellyfish *Aurelia* and *Clytia*. Our work demonstrates the shared ancestry of PNC1 and NAMPT pathways, suggesting that the lessons learned from model organisms using PNC1 could lead to new methods for boosting NAD production in mammals. What role these survival and longevity proteins play in early-branching animals—and whether any of them are related to the unusual aging dynamics seen in these groups—remains to be seen, but there is no lack of candidate species for future research.

## Materials and Methods

### Data Collection

We began by querying NCBI for proteins using BLASTP v2.9.0+ (Camacho et al. 2009). For all sirtuin searches, we used a series of four queries: *Mus musculus* SIR4 (NCBI accession: Q8R216), *Nematostella vectensis* SIR1 (XP_001623784.3), *Amphimedon queenslandica* SIR5 (XP_003382458.1), and *Volvox carteri* SIR6 (XP_002950001.1). These queries were chosen for encompassing the diversity of species and sirtuins considered in this study. For NAMPT, we used the *Homo sapiens* protein (UniProtKB ID: P43490) and for PNC1, we used the protein from *Sa. cerevisiae* (UniProtKB ID: P53184) as queries. The following databases were sequentially queried on NCBI’s non-redundant protein database: (1) Opisthokonta (taxid:33154), excluding fungi (taxid:4751) and animals (taxid:33208), and (2) animals (taxid:33208) excluding bilaterians (taxid:33213). To study the evolution of PNC1 and NAMPT across invertebrates, we ran a third BLASTP search including animals (taxid:33208) but excluding vertebrates (taxid:7742). BLAST searches were performed using the default parameters (*e*-value 0.05 and word size 6), but we increased the maximum number of target sequences to 5,000 to ensure that we collected every potential sirtuin. All target sequences were downloaded for downstream analyses.

In addition to the searches on NCBI, we created a custom database, with the goal of sampling a comprehensive set of early-branching animals alongside a smaller, phylogenetically diverse sampling of outgroups. This database, collected from UniProt (Anon 2021), included the following proteomes: the brown algae *Ectocarpus siliculosus* (UniProt proteome ID: UP000002630); the diatom *Fistulifera solaris* (UP000198406); the green algae *V. carteri* (UP000001058); the stonewort plant *Chara braunii* (UP000265515); the fungi *Galerina marginata* (UP000027222), *Puccinia graminis* (UP000008783), *Rhizophagus irregularis* (UP000236242), *Rozella allomycis* (UP000030755), *Sa. cerevisiae* (UP000002311), *Schizosaccharomyces pombe* (UP000002485), and *Spizellomyces punctatus* (UP000053201); the animals *Capitella teleta* (UP000014760), *Caenorhabditis elegans* (UP000001940), and *Chiloscyllium punctatum* (UP000287033); *Ciona intestinalis* (UP000008144), *Daphnia magna* (UP000076858), *Drosophila melanogaster* (UP000000803), *H. sapiens* (UP000005640), *Latimeria chalumnae* (UP000008672), *M. musculus* (UP000000589), *Octopus bimaculoides* (UP000053454), *St. purpuratus* (UP000007110), and *Xenopus laevis* (UP000186698). Additional data sets were added to study clades that are poorly sampled in NCBI. To include a planarian, we queried the *Schmidtea mediterranea* proteome (‘SmedSxl Genome Annotations’ version 4.0) from SmedGD (http://smedgd.stowers.org, accessed September 5, 2020). To include data on jellyfish, we queried *Aurelia* “sp.1” (also known as *Aurelia coerulea*) using private protein models (provided on the GitHub repository), and *Clytia hemisphaerica* from the Marine Invertebrate Models Database (http://marimba.obs-vlfr.fr, accessed August 20, 2020). To increase our sampling of sponges, we queried the following proteomes on Compagen (http://compagen.org, accessed August 20, 2020): *Ephydatia muelleri*, *Haliclona amboinensis*, *Haliclona tubifera*, *Leucosolenia complicata*, *Oscarella carmela*, *Stylissa carteri*, and *Sycon coactum*. Since no ctenophore sirtuins were recovered from NCBI, we queried the proteome of *Mnemiopsis leidyi* through Ensembl Metazoa v.47 (https://metazoa.ensembl.org/, accessed August 20, 2020) and the unfiltered gene models of *Pleurobrachia bachei* through Neurobase (https://neurobase.rc.ufl.edu/, accessed August 20, 2020). BLASTP was run with default values for the standalone version (word_size = 3; *e*-value = 10), and all target sequences were retained for downstream analyses.

All fasta sequences from all searches were combined into a single file and reformatted so that each sequence ID began with the species name. We then performed a BLASTP search using our genes as queries against the UNIPROT/SWISSPROT data set of vetted genes. The top BLAST hit was appended to the end of each protein name. This data set is provided on GitHub as “3_Sirtuin_BLAST_Hits.Annotated.fasta.”

### Data Vetting

Conserved protein domain identification in the BLAST hits was performed using the PfamScan program in the EMBL-EBI toolkit (Li et al. 2015). The output from PfamScan was converted into GFF3 format using the pfam2gff.py script in genomeGTFtools (https://github.com/wrf/genomeGTFtools). For NAMPT and PNC1, conserved domains were extracted from the original fasta files using the GFF3 annotation file and SAMtools v1.9 (Danecek et al. 2021), based on the GFF3 “alignment start” and “alignment end” coordinates. For sirtuins, the domain extraction technique described above did not work, as the SIR2 domain (PFAM ID: PF02146) was often annotated as multiple fragments. Instead, the fasta file and GFF3 annotations were loaded into Geneious v9.1.8. The SIR2 PFAM domains were extracted from the sequences and used for subsequent analyses. We identified two proteins featuring two complete SIR2 domains (“Amphimedon_queenslandica|XP_019864326.1” and “Stylissa_carteri|maker-SC_scaffold3240-augustus-gene-0.61-mRNA-1”); these domains were divided into separate fasta sequences by hand.

To remove redundant results, the fasta files containing conserved domains were separated by species ID, and then run through CD-Hit v.4.8.1 (Fu et al. 2012). Conserved domains that were >95% identical were collapsed into the longest sequence. For the sirtuins, we performed an additional step removing any conserved domain shorter than 90 basepairs (less than half of the canonical domain). Following CD-Hit analysis the sequences from all species were concatenated back into individual fasta files for sirtuin, PNC1, and NAMPT data sets.

### Protein Alignment and Tree Building

For sirtuins, we tested several methods for protein alignment. We began with the sequence aligner PRANK, which has the benefit of using species relationships to infer insertion/deletion events (Löytynoja 2021). Unfortunately, the evolutionary distance between species and uncertainties in phylogeny/branch length prevented PRANK from producing meaningful results. The details of this analysis are provided on GitHub. We ultimately chose the E-INS-i method in MAFFT v7.487, which is designed for conserved motifs embedded in long, non-homologous regions (Katoh and Standley 2013). The phylogeny was produced using IQ-TREE v.1.6.12 (Nguyen et al. 2015) with 1,000 rapid bootstraps; we used the ModelFinder program packaged in IQ-TREE to choose the best-fit model of protein evolution (LG + R10). The MAFFT/IQ-TREE approach was used for the PNC1 and NAMPT trees. PNC1 genes were rooted with additional members of the cysteine hydrolase superfamily (PFAM ID: cd00431), while the NAMPT tree was rooted with domains from PRTase type II (PFAM ID: cd00516) and QPRTase NadC (PFAM ID: cd01568). The sirtuin tree was rooted with Class III based on the hypothesis, it represents the oldest clade (Frye 2006).

The sirtuin data set went through multiple rounds of tree building. After the first analysis, the resulting tree was examined for evidence of bacterial/archaeal contamination. The tree is reproduced in supplementary fig. S6, Supplementary Material online, and the probable contaminants are labeled in red. These sequences, listed in supplementary table S2, Supplementary Material online, were excluded from further analyses. After the second round of gene alignment and tree building, species-tree correction of the gene tree was performed using NOTUNG (Chen et al. 2000). This program rearranges branches on a gene tree with low node support to minimize the number of gene duplications and losses. We used a cutoff of 90% for node support. The resulting tree (file: 9b_Domain_NOTUNG_Final.tree) was used to produce figs. 2 and 3 in the main text. There is ongoing debate as to the relationships between ctenophores, sea sponges, and the other animals. Because NOTUNG does not allow for polytomies in the species tree, we initially ran our analysis with ctenophores as the basal animal group. To test whether a “basal-sponge” phylogeny would dramatically change our results, we re-ran the analysis with a different species tree. The results did not impact the relationships of the sirtuin families to each other, or the presence/absence of various sirtuins in animals. We provide this additional analysis on GitHub.

In addition to studying the entire sirtuin clade, we performed an analysis on Class SIR2. The list of SIR2 genes, as determined from the original analysis, were extracted from the original data set (file: 3_Sirtuin_BLAST_Hits.Annotated.fasta), and went through a separate round of MAFFT alignment, IQ-TREE tree building, and NOTUNG tree reconciliation. The results of this analysis were used to make fig. 5 and supplementary fig. S5, Supplementary Material online.

### Protein Modeling

Modeling of protein tertiary structure was performed in the I-TASSER webserver (Yang et al. 2015). Two proteins were submitted for analysis: SIR8 from the sea sponge *A. queenslandica* (NCBI accession: XP_011406188), and SIR2 from the yeast *Sa. cerevisiae* (NCBI accession: P06700.1). The results were visualized and studied in Open-Source PyMOL (https://github.com/schrodinger/pymol-open-source). The results from I-TASSER and the PyMOL code are provided on GitHub (folder: 3_I-TASSER_Results).

## Supplementary Material

msac192_Supplementary_Data

## Data Availability

The code used to produce this data and relevant input/output files are available on GitHub at https://github.com/davidgoldlab/2022_Sirtuin_Evolution.
