# Supplementary material for: Sirtuin Evolution at the Dawn of Animal Life
Source: Mol Biol Evol. 2022 Sep 6;39(9):msac192. doi: 10.1093/molbev/msac192 (PMC9486876; doi:10.1093/molbev/msac192)

SUPPLEMENTARY TABLES

Table S1. Additional conserved protein domains identified on sirtuin proteins in our dataset.

**Domain**	**Found in species**	**Sirtuin Clade**	**gene ID (short)**	**Position to SIR2 domain**
CAP_C	*Acropora millepora*	SIR8	XP_029214425.1	Upstream
CAP_C	*Actinia tenebrosa*	SIR8	XP_031565139.1	Upstream
CAP_C	*Amphimedon queenslandica*	SIR8	XP_011406188.1	Upstream
CAP_C	*Orbicella faveolata*	SIR8	XP_020631316.1	Upstream
CAP_C	*Pocillopora damicornis*	SIR8	XP_027046210.1	Upstream
CAP_C	*Stylophora pistillata*	SIR8	XP_022790004.1	Upstream
DUF592	*Puccinia graminis*	SIR1	KAA1082764.1	Upstream
DUF592	*Rhizophagus irregularis*	SIR1	PKC08513.1	Upstream
DUF592	*Saccharomyces cerevisiae*	SIR1	4IAO	Upstream
DUF592	*Saccharomyces cerevisiae*	SIR1	NP_014573.1	Upstream
DUF592	*Schizosaccharomyces pombe*	SIR1	NP_001018840.1	Upstream
Img2	*Dendronephthya gigantea*	SIR2	XP_028398492.1	Upstream
Mob_synth_C	*Pleurobrachia bachei*	SIR7	sb|2669295	Downstream
Oxysterol_BP	*Mnemiopsis leidyi*	SIR3	ML002624a	Upstream
Radical_SAM	*Pleurobrachia bachei*	SIR7	sb|2669295	Downstream

Table S2: Probable contaminating sequences / horizontal gene transfer events in our dataset. See Figure S6 for the relevant gene tree.

**Scientific name**	**gene ID (short)**	**Species with most similar protein**
Aurelia coerulea	TCONS_00008113_Seg122.19.p1	*Aquifex aeolicus*
Capitella teleta	ELT91158.1	*Methanopyrus kandleri*
Clytia hemisphaerica	TCONS_00049926	*Thermus thermophilus*
Leucosolenia complicata	lcpid67440_lcgid21395	*Aquifex aeolicus*
Salpingoeca rosetta	XP_004993987.1	*Pyrobaculum aerophilum*
Strongylocentrotus purpuratus	XP_030829218.1	*Caldanaerobacter subterraneus*
Stylissa carteri	scaffold70346-gene-0.28	*Thermococcus kodakarensis*
Sycon ciliatum	scpid89809_scgid18507	*Saccharolobus solfataricus*

SUPPLEMENTARY FIGURE LEGENDS

Figure S1: A cartoon demonstrating the difference between orthologs and paralogs, and why the distinction shifts at different levels of evolutionary analysis. (A) A hypothetical gene tree of “family 3” sirtuins, based on the results of Opazo et al. (2020) Note that the fish and frog have two copies of the gene, which are distinguished as “Sir3” and “Sir 3-like”. From the vantage of vertebrates, Sir3 and Sir3-like are paralogs of each other, as signified by the colored boxes. A scientist who assumes that, for example, “Frog Sir3-like” and “Mouse Sir3” play similar roles in their respective organisms is likely to generate a flawed hypothesis. However, from the vantage of all animals (including invertebrates like sea urchins and jellyfish), vertebrate Sir3 and Sir3-like are both orthologs of their invertebrate counterpart. (B) The gene tree described in (A) reconciled with the species tree. In this scenario, the two genes in fish and frogs originate from a single gene duplication event that occurred early in vertebrate history. The mouse lost one of these two paralogs over the course of evolution. It is worth noting that other scenarios of gene duplication and loss could explain the gene tree seen in (A).

Figure S2. The full phylogeny used to design Figure 2. Clades are colored based on their assignment in Figure 2. See GitHub file “9b_Domain_NOTUNG_Final.tree” for the relevant tree file.

Figure S3. Partial alignment of SIR6 and SIR8 sequences. A conserved nine amino acid motif is identified at the top of the figure, although additional differences between SIR6 and SIR8 exist. See GitHub file “9_SIR2_Domains.vetted.mafft.fasta” for the full data. Image made in Geneious v.9.1.8.

Figure S4. A partial protein alignment showing a unique SIR9 motif and its distribution across animals. Additional SIR9 proteins from animals that were not queried in the original analysis are shown at the bottom of the alignment. Image made in Geneious v.9.1.8.

Figure S5. The full phylogeny used to design Figure 5. See GitHub file “4_NOTUNG-SpongeFirst-Final_Tree.tree” for the relevant tree file.

Figure S6. Initial phylogeny of sirtuin protein domains in our study. Likely contaminants / possible horizontal gene transfers are colored red and were excluded from further analysis. See GitHub file “8_SIR2_Domains.mafft.fasta.contree.tree” for the relevant tree file.

Figure S7. The full phylogeny used to inform Figure 6. Probable losses of PNC1 and NAMPT are labeled on the tree.

Figure S1

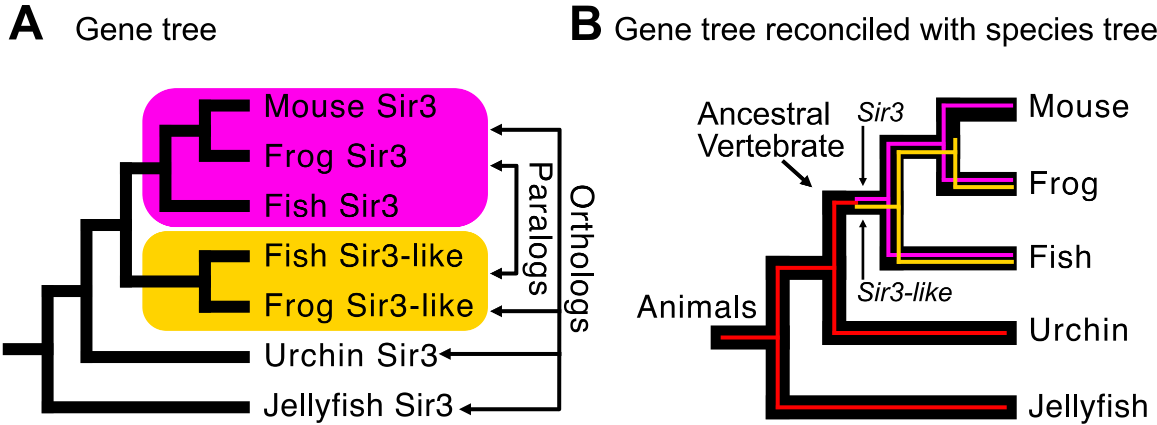
A
Gene tree
Mouse Sir3
Frog Sir3
Fish Sir3
Fish Sir3-like
Frog Sir3-like
Urchin Sir3
Jellyfish Sir3
Paralogs
Orthologs
B
Gene tree reconciled with species tree
Ancestral Vertebrate
Sir3
Sir3-like
Animals
Mouse
Frog
Fish
Urchin
Jellyfish

Figure S2

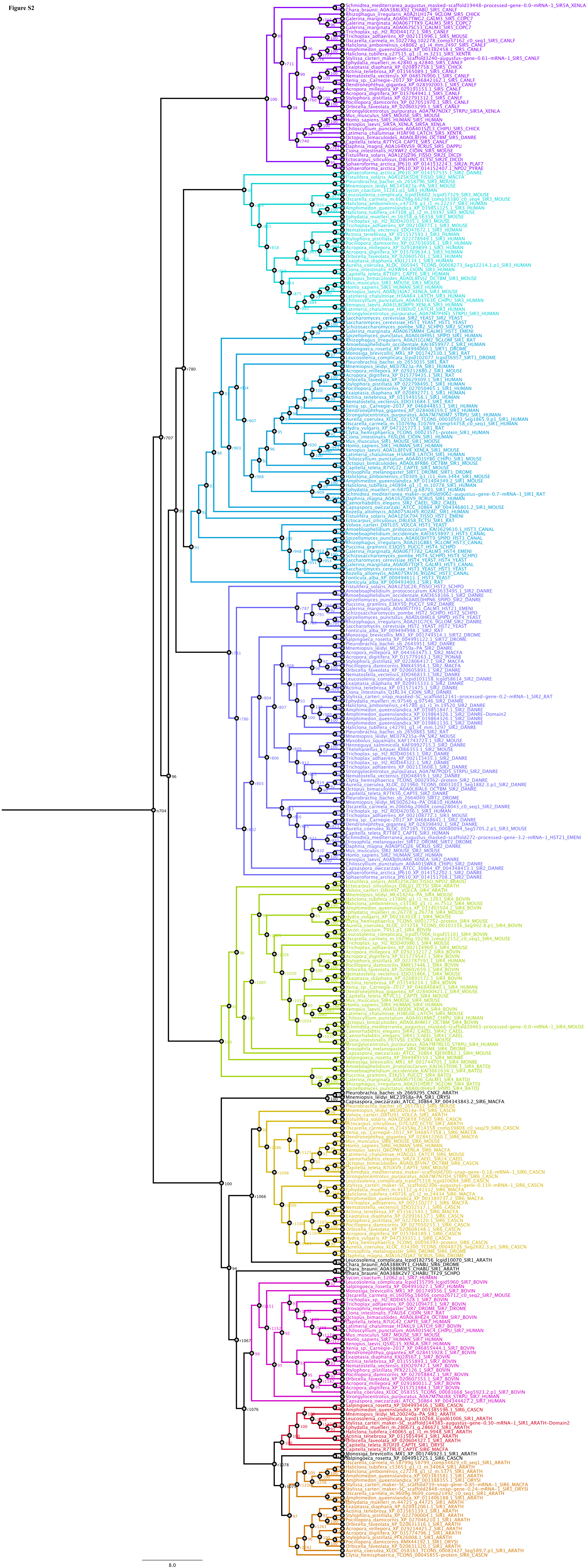

Figure S3

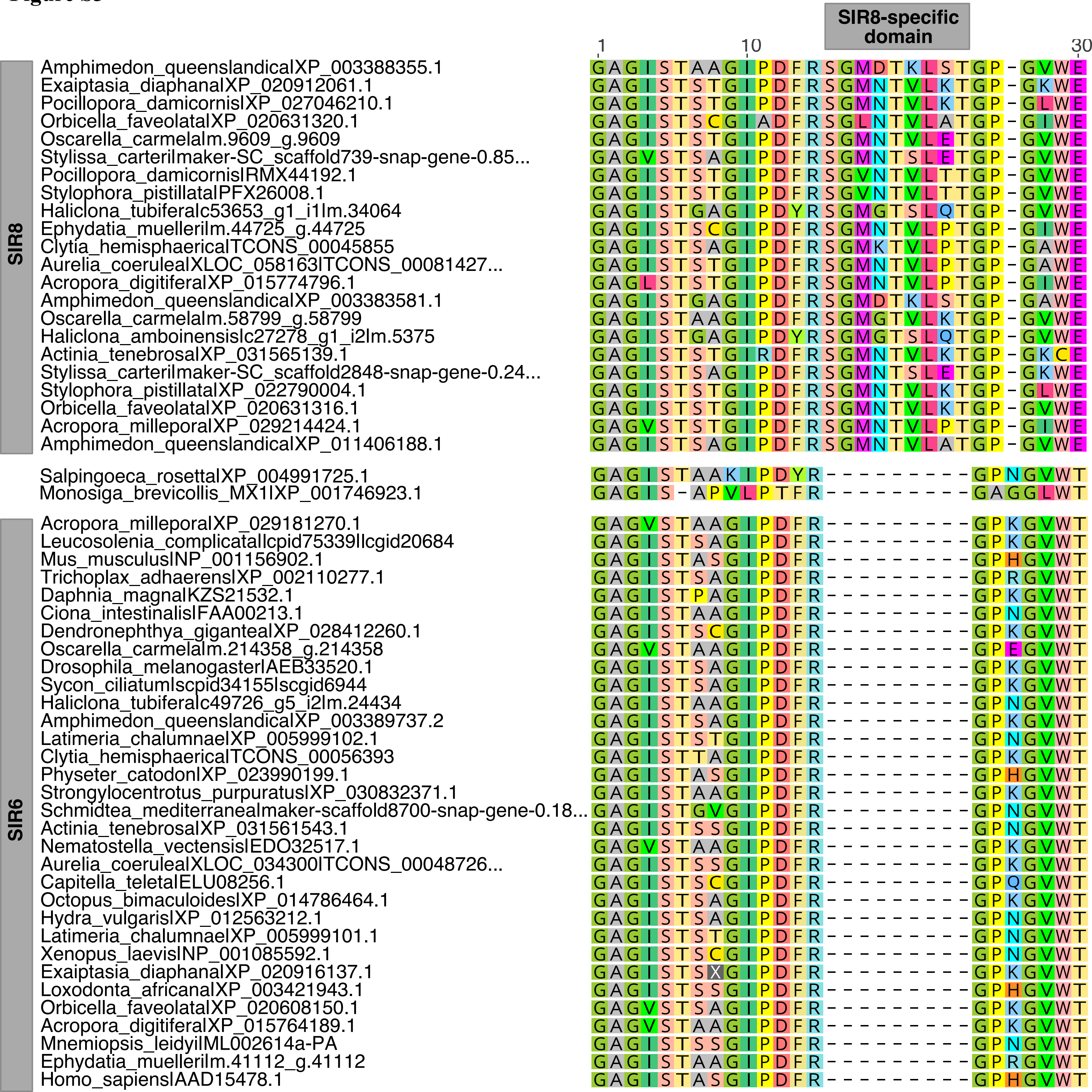

SIR8-specific domain
1
10
30
SIR8
SIR6
Amphimedon_queenslandica|XP_003388355.1
Exaiptasia_diaphana|XP_020912061.1
Pocillopora_damicornis|XP_027046210.1
Orbicella_faveolata|XP_020631320.1
Oscarella_carmela|m.9609_g.9609
Stylissa_carteri|maker-SC_scaffold739-snap-gene-0.85...
Pocillopora_damicornis|RMX44192.1
Stylophora_pistillata|PFX26008.1
Haliclona_tubifera|c53653_g1_i1|m.34064
Ephydatia_muelleri|m.44725_g.44725
Clytia_hemisphaerica|TCONS_00045855
Aurelia_coerulea|XLOC_058163|TCONS_00081427...
Acropora_digitifera|XP_015774796.1
Amphimedon_queenslandica|XP_003383581.1
Oscarella_carmela|m.58799_g.58799
Haliclona_amboinensis|c27278_g1_i2|m.5375
Actinia_tenebrosa|XP_031565139.1
Stylissa_carteri|maker-SC_scaffold2848-snap-gene-0.24...
Stylophora_pistillata|XP_022790004.1
Orbicella_faveolata|XP_020631316.1
Acropora_millepora|XP_029214424.1
Amphimedon_queenslandica|XP_011406188.1
Salpingoeca_rosetta|XP_004991725.1
Monosiga_brevicollis_MX1|XP_001746923.1
Acropora_millepora|XP_029181270.1
Leucosolenia_complicata|lcpid75339|lcgid20684
Mus_musculus|NP_001156902.1
Trichoplax_adhaerens|XP_002110277.1
Daphnia_magna|KZS21532.1
Ciona_intestinalis|FAA00213.1
Dendronephthya_gigantea|XP_028412260.1
Oscarella_carmela|m.214358_g.214358
Drosophila_melanogaster|AEB33520.1
Sycon_ciliatum|scpid34155|scgid6944
Haliclona_tubifera|c49726_g5_i2|m.24434
Amphimedon_queenslandica|XP_003389737.2
Latimeria_chalumnae|XP_005999102.1
Clytia_hemisphaerica|TCONS_00056393
Physeter_catodon|XP_023990199.1
Strongylocentrotus_purpuratus|XP_030832371.1
Schmidtea_mediterranea|maker-scaffold8700-snap-gene-0.18...
Actinia_tenebrosa|XP_031561543.1
Nematostella_vectensis|EDO32517.1
Aurelia_coerulea|XLOC_034300|TCONS_00048726...
Capitella_teleta|ELU08256.1
Octopus_bimaculoides|XP_014786464.1
Hydra_vulgaris|XP_012563212.1
Latimeria_chalumnae|XP_005999101.1
Xenopus_laevis|NP_001085592.1
Exaiptasia_diaphana|XP_020916137.1
Loxodonta_africana|XP_003421943.1
Orbicella_faveolata|XP_020608150.1
Acropora_digitifera|XP_015764189.1
Mnemiopsis_leidyi|ML002614a-PA
Ephydatia_muelleri|m.41112_g.41112
Homo_sapiens|AAD15478.1

Figure S4

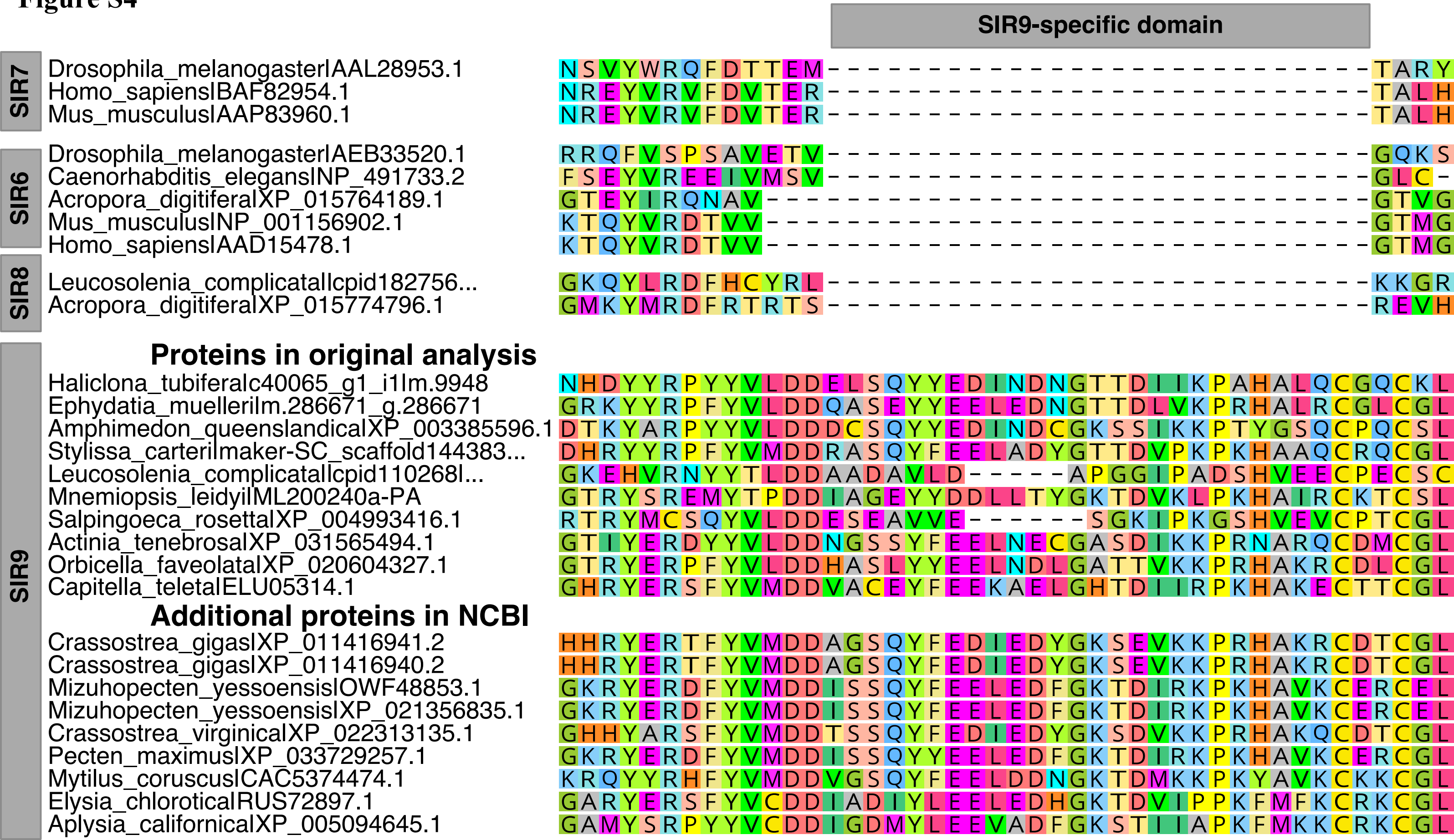

SIR9-specific domain
SIR7
Drosophila_melanogaster|AAL28953.1 NSVYWRQFDTTEM------------------TARY
Homo_sapiens|BAF82954.1 NREYVRVFDVTER------------------TALH
Mus_musculus|AAP83960.1 NREYVRVFDVTER------------------TALH
SIR6
Drosophila_melanogaster|AEB33520.1 RRQFVSPSAVETV------------------GQKS
Caenorhabditis_elegans|NP_491733.2 FSEYVREEIVMSV------------------GLC-
Acropora_digitifera|XP_015764189.1 GTEYIRQNAV---------------------GTVG
Mus_musculus|NP_001156902.1 KTQYVRDTVV---------------------GTMG
Homo_sapiens|AAD15478.1 KTQYVRDTVV---------------------GTMG
SIR8
Leucosolenia_complicata|lcpid182756... GKQYLRDFHCYRL------------------KKGR
Acropora_digitifera|XP_015774796.1 GMKYMRDFRTRTS------------------REVH
SIR9
Proteins in original analysis
Haliclona_tubifera|c40065_g1_i1|m.9948 NHDYYRPYYVLDDELSQYYEDINDNGTTDIIKPAHALQCGQCKL
Ephydatia_muelleri|m.286671_g.286671 GRKYYRPFYVLDDQASEYYEELEDNGTTDLVKPRHALRCGLCGL
Amphimedon_queenslandica|XP_003385596.1 DTKYARPYYVLDDDCSQYYEDINDCGKSSIKKPTYGSQCPQCSL
Stylissa_carteri|maker-SC_scaffold144383... DHRYYRPFYVMDDRASQYFEELADYGTTDVPKPKHAAQCRQCGL
Leucosolenia_complicata|lcpid110268|... GKEHVRNYYTLDDAADAVLD------APGGIPADSHVEECPECSC
Mnemiopsis_leidyi|ML200240a-PA GTRYSREMYTPDDIAGEYYDDLLTYGKTDVKLPKHAIRCKTCSL
Salpingoeca_rosetta|XP_004993416.1 RTRYMCSQYVLDDESEAVVE-------SGKIPKGSHVEVCPTCGL
Actinia_tenebrosa|XP_031565494.1 GTIYERDYYVLDDNGSSYFEELNECGASDIKKPRNARQCDMCGL
Orbicella_faveolata|XP_020604327.1 GTRYERPFYVLDDHASLYYEELNDLGATTVKKPRHAKRCDLCGL
Capitella_teleta|ELU05314.1 GHRYERSFYVMDDVACEYFEEKAELGHTDIIRPKHAKECTTCGL
Additional proteins in NCBI
Crassostrea_gigas|XP_011416941.2 HHRYERTFYVMDDAGSQYFEDIEDYGKSEVKKPRHAKRCDTCGL
Crassostrea_gigas|XP_011416940.2 HHRYERTFYVMDDAGSQYFEDIEDYGKSEVKKPRHAKRCDTCGL
Mizuhopecten_yessoensis|OWF48853.1 GKRYERDFYVMDDISSQYFEELEDFGKTDIRKPKHAVKCERCEL
Mizuhopecten_yessoensis|XP_021356835.1 GKRYERDFYVMDDISSQYFEELEDFGKTDIRKPKHAVKCERCEL
Crassostrea_virginica|XP_022313135.1 GHHYARSFYVMDDTSSQYFEDIEDYGKSDVKKPRHAKQCDTCGL
Pecten_maximus|XP_033729257.1 GKRYERDFYVMDDISSQYYEELEDFGKTDIRKPKHAVKCERCGL
Mytilus_coruscus|CAC5374474.1 KRQYYRHFYVMDDVGSQYFEELDDNGKTDMKKPKYAVKCKKCGL
Elysia_chlorotica|RUS72897.1 GARYERSFYVCDDIADIYLEELEDHGKTDVIPPKFMFKCRKCGL
Aplysia_californica|XP_005094645.1 GAMYSRPYYVCDDIGDMYLEEVADFGKSTIIAPKFMKKCRKCGL

Figure S5

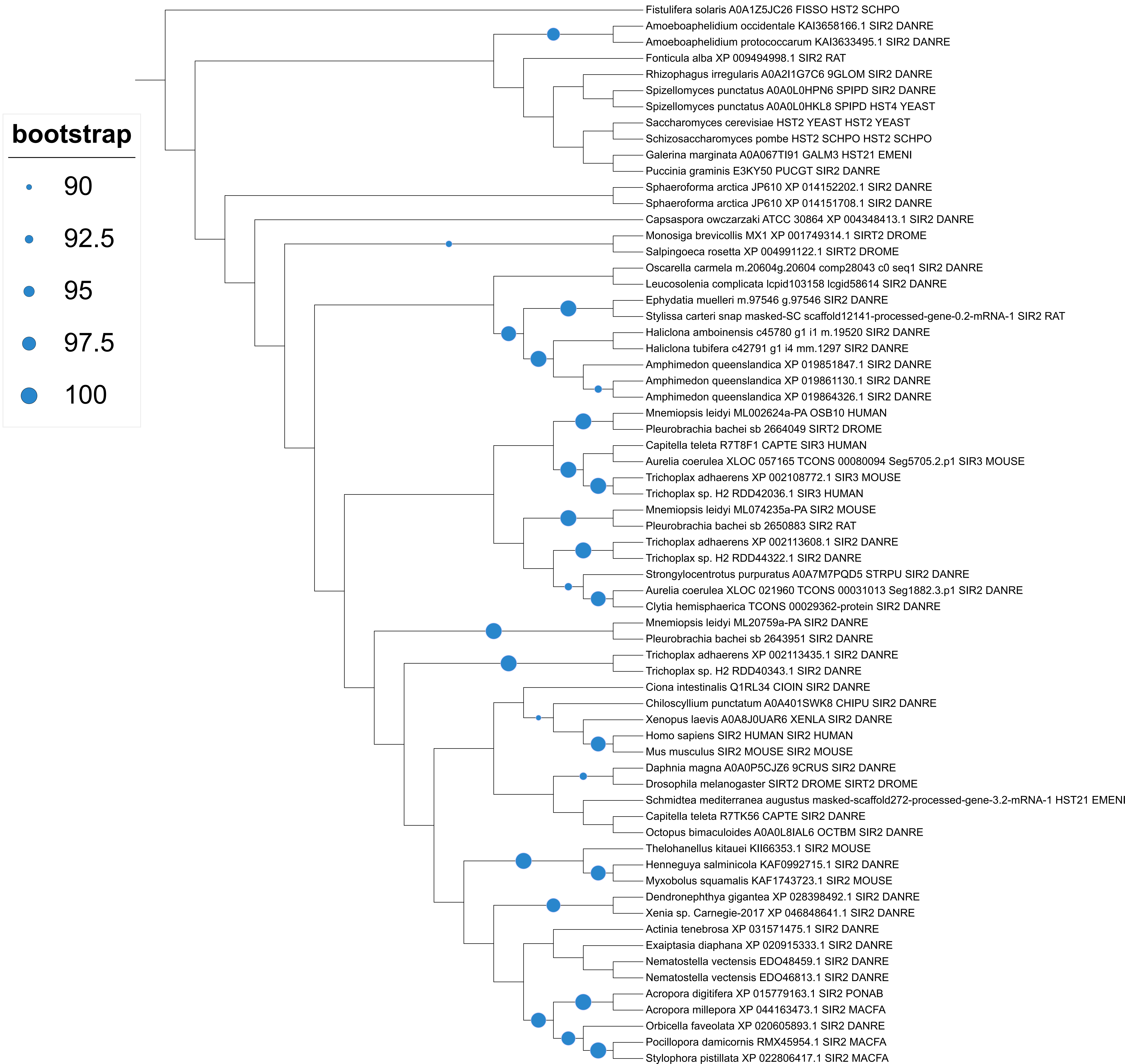
bootstrap
90
92.5
95
97.5
100
Fistulifera solaris A0A1Z5JC26 FISSO HST2 SCHPO
Amoeboaphelidium occidentale KAI3658166.1 SIR2 DANRE
Amoeboaphelidium protococcarum KAI3633495.1 SIR2 DANRE
Fonticula alba XP 009494998.1 SIR2 RAT
Rhizophagus irregularis A0A2I1G7C6 9GLOM SIR2 DANRE
Spizellomyces punctatus A0A0L0HPN6 SPIPD SIR2 DANRE
Spizellomyces punctatus A0A0L0HKL8 SPIPD HST4 YEAST
Saccharomyces cerevisiae HST2 YEAST HST2 YEAST
Schizosaccharomyces pombe HST2 SCHPO HST2 SCHPO
Galerina marginata A0A067TI91 GALM3 HST21 EMENI
Puccinia graminis E3KY50 PUCGT SIR2 DANRE
Sphaeroforma arctica JP610 XP 014152202.1 SIR2 DANRE
Sphaeroforma arctica JP610 XP 014151708.1 SIR2 DANRE
Capsaspora owczarzaki ATCC 30864 XP 004348413.1 SIR2 DANRE
Monosiga brevicollis MX1 XP 001749314.1 SIRT2 DROME
Salpingoeca rosetta XP 004991122.1 SIRT2 DROME
Oscarella carmela m.20604g.20604 comp28043 c0 seq1 SIR2 DANRE
Leucosolenia complicata lcpid103158 lcgid58614 SIR2 DANRE
Ephydatia muelleri m.97546 g.97546 SIR2 DANRE
Stylissa carteri snap masked-SC scaffold12141-processed-gene-0.2-mRNA-1 SIR2 RAT
Haliclona amboinensis c45780 g1 i1 m.19520 SIR2 DANRE
Haliclona tubifera c42791 g1 i4 mm.1297 SIR2 DANRE
Amphimedon queenslandica XP 019851847.1 SIR2 DANRE
Amphimedon queenslandica XP 019861130.1 SIR2 DANRE
Amphimedon queenslandica XP 019864326.1 SIR2 DANRE
Mnemiopsis leidyi ML002624a-PA OSB10 HUMAN
Pleurobrachia bachei sb 2664049 SIRT2 DROME
Capitella teleta R7T8F1 CAPTE SIR3 HUMAN
Aurelia coerulea XLOC 057165 TCONS 00080094 Seg5705.2.p1 SIR3 MOUSE
Trichoplax adhaerens XP 002108772.1 SIR3 MOUSE
Trichoplax sp. H2 RDD42036.1 SIR3 HUMAN
Mnemiopsis leidyi ML074235a-PA SIR2 MOUSE
Pleurobrachia bachei sb 2650883 SIR2 RAT
Trichoplax adhaerens XP 002113608.1 SIR2 DANRE
Trichoplax sp. H2 RDD44322.1 SIR2 DANRE
Strongylocentrotus purpuratus A0A7M7PQD5 STRPU SIR2 DANRE
Aurelia coerulea XLOC 021960 TCONS 00031013 Seg1882.3.p1 SIR2 DANRE
Clytia hemisphaerica TCONS 00029362-protein SIR2 DANRE
Mnemiopsis leidyi ML20759a-PA SIR2 DANRE
Pleurobrachia bachei sb 2643951 SIR2 DANRE
Trichoplax adhaerens XP 002113435.1 SIR2 DANRE
Trichoplax sp. H2 RDD40343.1 SIR2 DANRE
Ciona intestinalis Q1RL34 CIOIN SIR2 DANRE
Chiloscyllium punctatum A0A401SWK8 CHIPU SIR2 DANRE
Xenopus laevis A0A8J0UAR6 XENLA SIR2 DANRE
Homo sapiens SIR2 HUMAN SIR2 HUMAN
Mus musculus SIR2 MOUSE SIR2 MOUSE
Daphnia magna A0A0P5CJZ6 9CRUS SIR2 DANRE
Drosophila melanogaster SIRT2 DROME SIRT2 DROME
Schmidtea mediterranea augustus masked-scaffold272-processed-gene-3.2-mRNA-1 HST21 EMENI
Capitella teleta R7TK56 CAPTE SIR2 DANRE
Octopus bimaculoides A0A0L8IAL6 OCTBM SIR2 DANRE
Thelohanellus kitauei KII66353.1 SIR2 MOUSE
Henneguya salminicola KAF0992715.1 SIR2 DANRE
Myxobolus squamalis KAF1743723.1 SIR2 MOUSE
Dendronephthya gigantea XP 028398492.1 SIR2 DANRE
Xenia sp. Carnegie-2017 XP 046848641.1 SIR2 DANRE
Actinia tenebrosa XP 031571475.1 SIR2 DANRE
Exaiptasia diaphana XP 020915333.1 SIR2 DANRE
Nematostella vectensis EDO48459.1 SIR2 DANRE
Nematostella vectensis EDO46813.1 SIR2 DANRE
Acropora digitifera XP 015779163.1 SIR2 PONAB
Acropora millepora XP 044163473.1 SIR2 MACFA
Orbicella faveolata XP 020605893.1 SIR2 DANRE
Pocillopora damicornis RMX45954.1 SIR2 MACFA
Stylophora pistillata XP 022806417.1 SIR2 MACFA

Figure S6

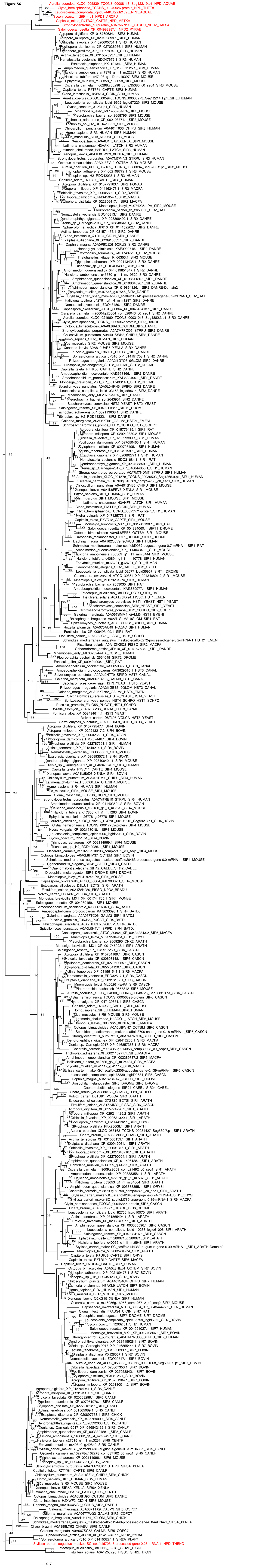

Figure S7

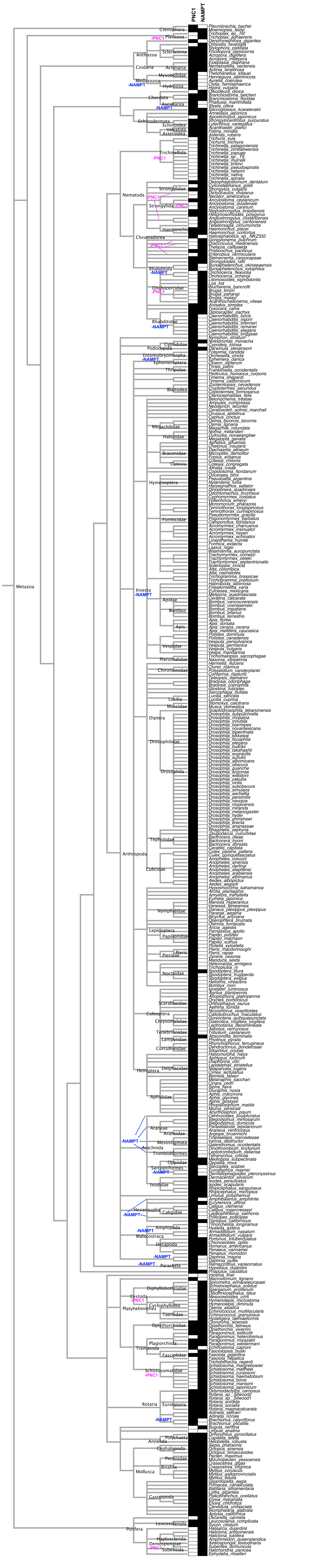

Supplement: msac192_Supplementary_Data [file msac192_supplementary_data.pdf]
